# Hypophosphites in Tuberculosis

**Published:** 1859-09

**Authors:** 


### HYPOPHOSPHITES IN TUBERCULOSIS.

We desire to direct the special attention of our readers to the remarks contained in this No. on Dr. Churchill's recommendation of the Hypophosphites in Tuberculosis. The reported *results* of the use of these agents, whether the theory connected with their recommendation be correct or not, should commend them to our attention as worthy of investigation.

